# Adult cancer survivors’ perceptions of immersive virtual reality exercise and its utility during chemotherapy infusion: a concurrent mixed method exploratory study

**DOI:** 10.1007/s00520-026-10920-7

**Published:** 2026-07-04

**Authors:** Katia Ferrar, Belinda Lange, Thomas Beltrame, Elizabeth S. Buckley, Keng Hao Chew, Jonathan J. Foo, Max K. Hollis, Emma Kemp, Bogda Koczwara, Peggy Lim, David Mizrahi, Nicole May, Jessica Thomson, Joan Schumacher, David Hobbs

**Affiliations:** 1 Flinders Health and Medical Research Institute, College of Medicine and Public Health, Flinders University, Sturt Road, Bedford Park 5042, Adelaide, SA Australia; 2 Australian Centre for Health Services Innovation and Centre for Healthcare Transformation, School of Public Health and Social Work, Queensland University of Technology, Brisbane, QLD Australia; 3 College of Health and Enablement, Flinders University, Adelaide, SA Australia; 4 College of Science and Engineering, Flinders University, Adelaide, South Australia Australia; 5 Interdisciplinary Graduate Program - Global Asia, Nanyang Technological University, Singapore, Singapore; 6 Vue Networks, Singapore, Singapore; 7 School of Science and Technology, Singapore University of Social Sciences, Singapore, Singapore; 8 Australian Research Centre for Cancer Survivorship, University of New South Wales, Sydney, NSW Australia; 9 Daffodil Centre, Sydney, NSW Australia; 10 Flinders Infusion Suite, Flinders Medical Centre, Adelaide, South Australia Australia; 11 Grampians Health, Ballarat, VIC Australia

**Keywords:** Virtual reality, Physical activity, Exercise, Cancer survivors, Chemotherapy

## Abstract

**Purpose:**

Virtual reality (VR) exercise may help promote physical activity in cancer survivors. Understanding infusion-specific barriers and facilitators is essential for designing effective interventions that can be integrated into chemotherapy care. This study explored adult cancer survivors’ perceptions and preferences regarding VR exercise during chemotherapy infusion.

**Methods:**

This exploratory concurrent mixed-methods study included community-dwelling adults (≥ 18 years) who had completed primary cancer treatment involving chemotherapy. Participants trialled a commercially available immersive VR exercise program paired with a sensor and seated pedal unit, completed a survey on digital technology confidence and preferences, and took part in a focus group or interview to discuss VR exercise during infusion. Qualitative content analysis was used to analyse transcripts.

**Results:**

Eight cancer survivors (6 female; mean age 63.6 ± 8.3 years) participated. Most reported being confident (87%) and comfortable (75%) using digital technologies. All participants enjoyed the VR exercise and indicated they would have used it during chemotherapy if available. Positive perceptions included mental wellbeing benefits, distraction, relaxation, and potential enhancement of drug circulation. Participants also identified negative aspects of the experience and potential barriers to implementation, such as cold caps, risk of cannula dislodgement, space constraints, and infusion‑chair suitability.

**Conclusion:**

This study provides the first insights into cancer survivors’ perceptions of immersive VR exercise during chemotherapy infusion. Findings highlight both the promise and practical considerations of integrating VR exercise into infusion care and represent an initial step toward developing a tailored VR exercise intervention for use during chemotherapy.

**Supplementary Information:**

The online version contains supplementary material available at 10.1007/s00520-026-10920-7.

## Introduction

Physical activity, including exercise, has demonstrable evidence to be an effective strategy in reducing the detrimental effects of cancer and treatment and consequently improving survivors’ health and wellbeing [1–4]. Physical activity during active treatment is recommended by the American Society of Clinical Oncology [5]. Despite the benefits, for some cancer types such as breast, prostate and colorectal cancer, less than 35% of cancer survivors meet physical activity recommendations [6, 7]. Physical activity levels can fluctuate over the disease trajectory, tending to reduce during the months while people receive anticancer infusion treatment [8, 9]. Levels do increase post treatment, but often do not return to pre-diagnosis levels [8]. Barriers to physical activity among cancer survivors include fatigue, lack of time, lack of education provided by health professionals, and fear of exercise [10, 11].

Experiences and attitudes of exercise and physical activity can also influence physical activity participation. How people feel during exercise predicts future physical activity engagement [12]. Enjoyment during an acute bout of exercise has been shown to be predictive of future physical activity participation [13]. Thus, strategies to increase enjoyment during exercise could be an effective strategy to promote habitual physical activity. Virtual reality (VR) has been proposed as one strategy to improve physical activity experiences. In the general population, immersive VR strategies (i.e. using head-mounted displays and motion controllers) have positively influenced enjoyment during exercise, compared to exercise with no VR or non-immersive VR (i.e. VR scene played on a standard computer or phone display and using a mouse or keyboard) [14].

Cancer survivors receiving chemotherapy typically attend infusion suites as frequently as weekly, with sessions lasting from 20 min to several hours. As such, chemotherapy infusion sessions are an opportunity to engage cancer survivors in supportive care interventions. Exercise during chemotherapy infusion has been explored in a small number of feasibility studies and appears safe and feasible [15–17]. Virtual reality interventions have been trialled during chemotherapy infusion, and although the quality of current evidence is low, VR shows promise as a distraction intervention to reduce chemotherapy symptoms and anxiety [18]. However, no studies have explored delivering an exercise session using VR during anticancer therapy infusion.

Identification of infusion-specific VR exercise barriers and facilitators is important to design effective and sustainable interventions that can be integrated into clinical care. This study aimed to explore the preferences and perceptions of VR exercise during infusion among adult cancer survivors who had received chemotherapy as part of their cancer treatment. The study also explored how participants typically spent their time during infusion sessions and their patterns of digital technology use.

## Method

### Design

The study was an exploratory concurrent mixed method study, approved by the Flinders University Human Research Ethics Committee (Project #7414). Reporting of the study was guided by the STROBE (cross-sectional) and COREQ guidelines [19, 20].

### Participants and recruitment

A convenience sample of community-dwelling adults (18 years or older) who had completed primary active cancer treatment and who had previously undergone chemotherapy infusion was invited to participate. All participants provided written informed consent. As the data collection session involved trialling an immersive VR exercise experience, the following exclusion criteria applied: (a) diagnosed with epilepsy or recent history of seizures (within past 6 months), (b) severe light sensitivity, (c) severe visual impairment, (d) impairment of the lower limbs that would limit the ability to pedal, (e) chronic diseases that could be exacerbated or complicated by exercise (e.g. uncontrolled cardiac conditions, chronic obstructive pulmonary disease), (f) bony metastases in the lower limbs or pelvis, (g) acute illness or (h) inability to perform light intensity exercise for any reason. Recruitment flyers and information were distributed via targeted social media, local cancer support groups, local private cancer-specific allied health therapy clinics and professional networks. As this study examines an area that has not previously been investigated, the purpose is exploratory rather than confirmatory. As such, a small sample size (*n* ~ 10) was deemed appropriate for generating initial insights.

### Study procedures

This study involved workshops where participants trialled a commercially available VR exercise software in combination with a motion sensor and seated pedal unit in a university laboratory and a cancer charity meeting room setting and discussed their perceptions of VR exercise being offered during chemotherapy infusion. Participants also completed a hard copy/paper questionnaire regarding digital technology preferences and proficiency. No others were present during the testing apart from the researchers and participants. In this concurrent mixed method exploratory study, the content analysis qualitative component was used to gain significant insight into a previously unexplored research area, while the quantitative component allowed recording of VR-related symptoms and digital technology status using standardised or previously used surveys, allowing description of the sample and comparison with previous studies. Participants were provided with a $10 voucher in recognition for their contribution.

Questionnaire items pertained to participants’ (a) sociodemographic data (age, gender, education, cultural identity), (b) clinical characteristics (cancer type, time since diagnosis, time since completion of chemotherapy and average infusion duration), (c) digital technology confidence (measured using a single item 5-point Likert scale from very poor to very good), (d) digital technology use (including device access, device use, and social media application use), (e) digital technology comfort (measured using a series of 16 questions (Likert score with 5 categories: strongly disagree to strongly agree), (f) activity frequency of 14 activities, e.g. email writing (frequency options of: at least once per week, at least once per fortnight, at least once per month, less often than once per month and never), (g) experience using input devices e.g. computer mouse (measured on a 5-point scale from no experience to a lot of experience), (h) need for digital technology assistance (yes or no) and (i) activities engaged during chemotherapy infusion. The digital technology items were survey questions previously developed and used by one of the research team [21].

To capture participants’ experiences of VR exercise, participants completed the Simulator Sickness Questionnaire [22], which assesses the virtual environment’s effect on 16 symptoms such as eye strain and nausea (rated as none, slight, moderate and severe), before and after the VR exercise bout. After completing the VR exercise bout, participants also completed the System Usability Scale [23], a 10-item scale (5-point Likert: strongly agree–strongly disagree) giving a global view of subjective assessments of usability, and the Presence Questionnaire [24], a 6-item scale (7-point Likert) capturing the level of presence in immersive virtual environments.

### Immersive VR exercise experience

**Fig. 1:**
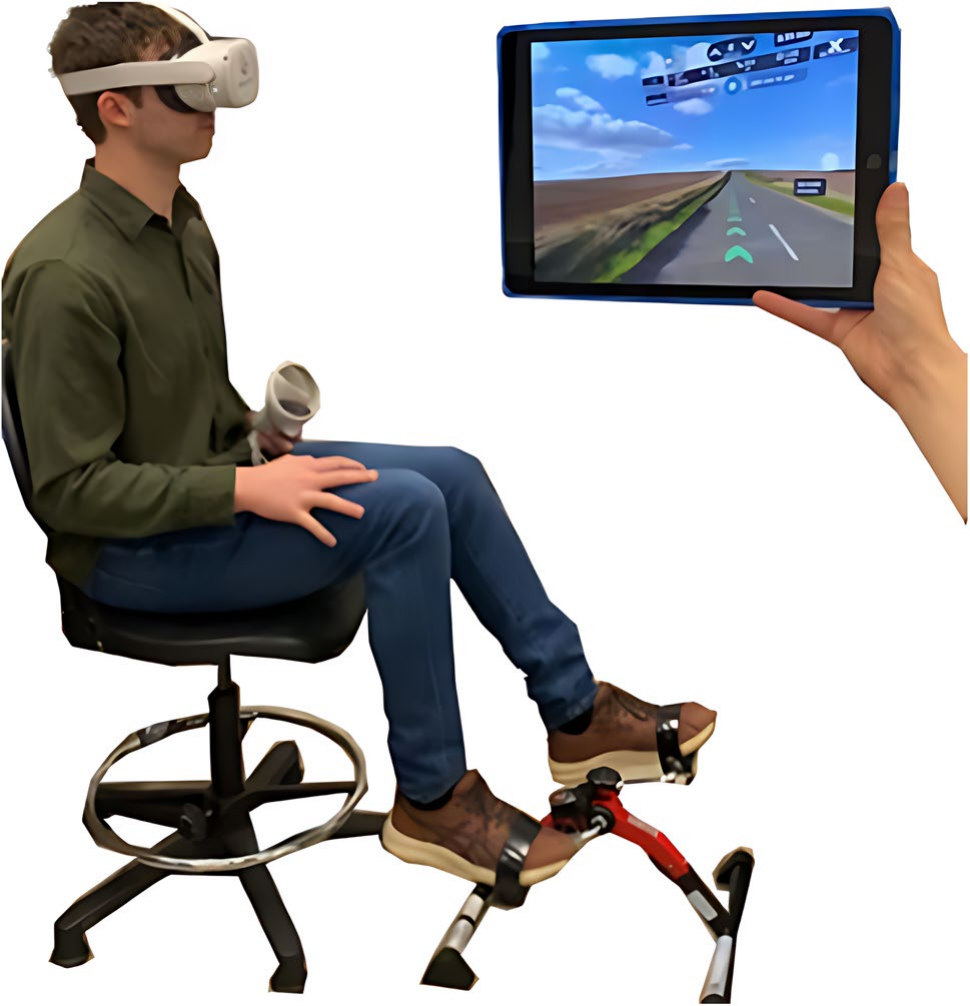
Immersive virtual reality seated pedal exercise experience set up (image supplied by authors with consent)

Participants engaged in VR exercise using a Meta Quest 2 headset (Meta, USA), VR exercise software (VZFit: VirZOOM Inc. USA), a commercially available seated pedal unit (Max Mobility Pedal Exerciser, PE1, Max Healthcare Equipment, Australia) and a motion sensor (i.e. accelerometer) attached to the pedal crank (VirZOOM Inc., VZ Sensor-1) (Fig. 1). Various cycling routes could be sampled in the VZFit software. Due to software issues, two participants experienced animated environments in VZFit, with the remainder experiencing a more real-life environment constructed from Google map images, and one participant experienced limited virtual travel due to pedal sensor issues. Participants cycled for approximately 5–10 min depending on their preference. The exertion level of the cycling activity was limited to 11 or less on the Borg 6–20 Ratings of Perceived Exertion Scale, equivalent to light intensity exercise [25]. Participants could stop the VR exercise bout at any time.

Once participants had completed the VR exercise bout, they were either interviewed individually or participated in a focus group discussion (conducted by author KF (female Senior Research Fellow; PhD), and assisted on two occasions by author BL (female Associate Professor; PhD), and one occasion by author TB (male Biomedical Engineering graduate; PhD candidate) exploring their perceptions of the experience and their thoughts on its potential for use during chemotherapy infusion. An interview guide developed for this study was used. Interviewer KF, a registered physiotherapist and researcher, was trained and experienced in interview and focus group facilitation. Interviews and focus group discussions were audio recorded. No relationship was established with participants prior to study commencement, and participants were provided with author KF’s reasons for conducting the research.

### Data analysis

Questionnaire data are presented as frequencies or means (standard deviation, SD). Digital comfort was summarised as favourable, neutral or unfavourable, determined by whether the majority of Likert responses were positive, neutral or negative, respectively. For the System Usability Scale (SUS), to get the overall SUS score, sum of the item score contributions was multiplied by 2.5, to produce a score from 0 (very poor perceived usability) to 100 (excellent perceived usability) in 2.5-point increment, with an average experience = 60 and a good experience = 80. For the Simulator Sickness Questionnaire, a total score (post-pre scores) was calculated as (Nausea subscale + Oculomotor subscale + Disorientation subscale) × 3.74, with higher scores indicating greater symptom severity, with severity considered minimal if the total score is less than 10. With the Presence Questionnaire, scores 1–7 were summed for each item, and total item score was then divided by 6. Group and individual discussion audio files were transcribed verbatim and sent to all participants for member checking. None of the participants requested amendments to the content of the transcripts (except one typographical error). Given the nature of the research question, to capture what was said at a descriptive level, qualitative content analysis, which allows for both descriptive and interpretive analysis, was used [26]. Meaning units, codes and categories were developed, and no themes were identified. One coder coded the data.

## Results

Eight cancer survivors (6 females) volunteered for the study. Data on participant sociodemographic profile, digital technology use and preferences and use of time during past chemotherapy sessions are provided in Table 1. All participants reported using a mobile phone daily and none had experience using VR. Most participants reported being confident (87%) and comfortable (75%) using digital technologies.

**Table 1:** Participant characteristics

Characteristics (*n* = 8)		
Age, mean (SD)		63.6 (8.3)
Gender, *n* (% sample)	Male	2 (25)
	Female	6 (75)
	Non-binary	0 (0)
Highest education, *n* (% sample)	Completed secondary school	0 (0)
	Trade qualification	2 (25)
	Bachelor’s degree	3 (38)
	Postgraduate degree	2 (25)
	Doctorate	1 (13)
Cultural identity, *n* (% sample)	Anglo Saxon	1 (13)
	Australian	5 (63)
	Australian Chinese	1 (13)
	Don’t know/prefer not to say	1 (13)
Years since cancer diagnosis, mean (SD)		4.9 (4.3)
Years since chemotherapy completion, mean (SD)		3.4 (4.5)
Average chemotherapy session duration, hrs (SD)		3.5 (1.6)
Digital technology confidence, *n* (% sample)	Very poor/poor	0 (0)
	Fair	1 (13)
	Good/very good	7 (87)
Digital technology comfort, *n* (% sample)	Favourable	6 (75)
	Neutral	1 (13)
	Unfavourable	1 (13)
Activities during chemotherapy infusion,* n* (% sample)	Reading	6 (75)
	Talking/socialising	6 (75)
	Watching movies	6 (75)
	Sleeping	5 (63)
	Emails	4 (50)
	Listening to music	4 (50)
	Searching internet	4 (50)
	Social media	3 (38)
	Podcasts	3 (38)
	Meditating	2 (25)
	Working	1 (13)
	Computer games	1 (13)
	Other (television)	1 (13)

*SD* standard deviation

### Virtual reality experience-related survey data

Adverse events captured by the Simulator Sickness Questionnaire suggest minimal intensity symptoms with a mean = 8.42 (Table 2). System Usability Score was average at a mean score of 67.81. Presence was overall moderate with a mean score of 4.33.

**Table 2:** Virtual reality experience-related survey data

Simulator Sickness Questionnaire	
Post-pre range	-11.22 - 44.88
Mean (SD)	8.42 (16.82)
System Usability Scale	
Range	45–82.5
Mean (SD)	67.81 (15.4)
Presence Questionnaire	
Range	1.67–5.33
Mean (SD)	4.33 (1.3)

*SD* standard deviation

### Content analysis

Coding details are provided in Online Resource 1.

### Positive immersive VR exercise experiences

**Fig. 2:**
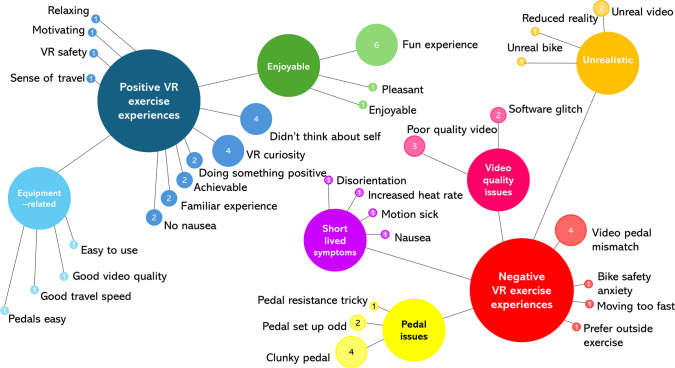
Content analysis findings for VR exercise experience. Categories (large circles with category title), sub-categories (medium circles with sub-category name) and codes (small circles, size relative to reported frequency, and n frequency reported in circle)

Responses from all participants suggested they enjoyed the VR experience. Beyond enjoyment, there was a sense of achievement and a feeling of doing something positive (Fig. 2). Several participants reported a positive outcome being that they didn’t think about themselves during the experience. For instance, participants reported:

Just your mind going somewhere else…. (70-year-old female breast cancer survivor)

So, you don’t think about yourself, you’ve got something else to think about. (68-year-old female breast cancer survivor)

Other positive aspects are related to ease of use of the equipment (headset and pedals) and the quality of the video experience.

### Negative immersive VR exercise experiences

Most of the participants identified some issues with the synchronisation between the video images and pedal action (Fig. 2). For example, one participant reported:

I was peddling fairly hard and getting a bit of resistance, but I wasn’t moving fast as I would have thought for that amount of energy. So that’s the disconnect – so matching those up is important. (female multiple myeloma cancer survivor)

Some participants reported issues with the equipment and video quality. The pedal action was not always smooth, and it was difficult to set the correct resistance level. Some participants reported the video quality made the experience less real. One participant reported “all the trees were distorted, so it didn’t quite look as real” (60-year-old male bladder cancer survivor). These experiences may have affected the level of presence and be reflected in the moderate presence score. Convergent with the quantitative symptom data, some participants also reported some short-lived symptoms such as a feeling of disorientation, nausea, motion sickness and increased heart rate.

**Fig. 3:**
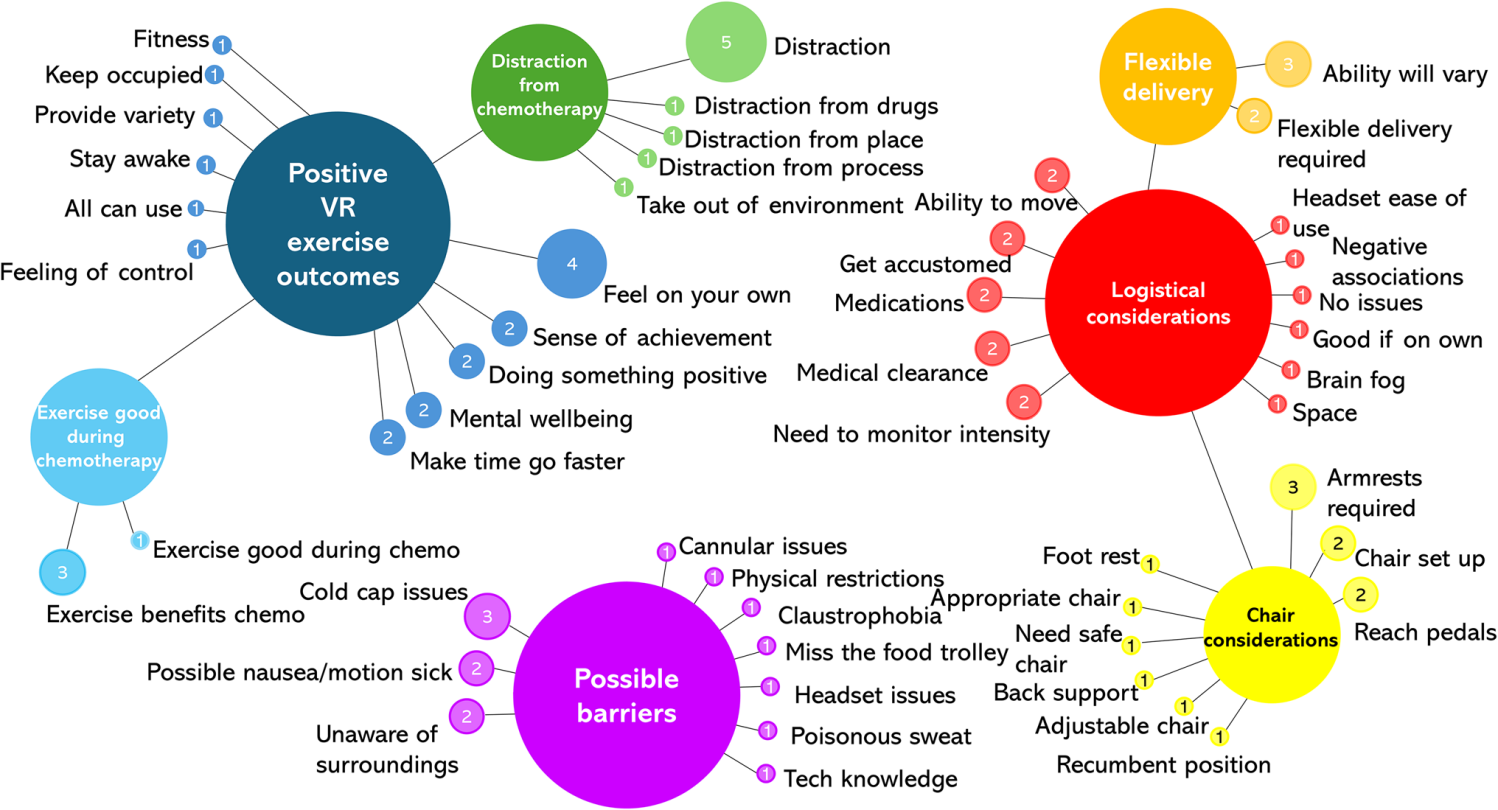
Content analysis findings for VR exercise utility. Categories (large circles with category title), sub-categories (medium circles with sub-category name) and codes (small circles, size relative to reported frequency, and *n* frequency reported in the circle)

Regarding the potential utility of the VR exercise experience during chemotherapy infusion, three categories developed including positive VR exercise outcomes, possible barriers and logistical considerations (Fig. 3).

### Positive VR exercise outcomes

Distraction from the chemotherapy environment was indicated by all participants as a potential benefit to cancer survivors undergoing chemotherapy (Fig. 3). More specifically, distraction from the chemotherapy infusion drugs, the infusion suite environment and the infusion suite processes were mentioned. The benefit of exercise to aid circulation of chemotherapy drugs was also suggested as a potential benefit. Other benefits included feeling on your own, a sense of achievement and the act of doing something positive.

### Possible barriers

The participants were encouraged to consider possible barriers (Fig. 3). Several participants flagged that participation for those using a cold cap may be a limitation. Other barriers included possible nausea or motion sickness, possible issues with cannula insertion safety (i.e. cannula dislodging) and possible claustrophobia. Several participants mentioned that it may be a problem if participants were unaware of their surroundings in the infusion suite. For example, one participant raised a concern about “Is someone going to take my stuff while I’m in VR?” (51-year-old female breast cancer survivor). Technical knowledge was only identified by one participant as a possible barrier, somewhat discordant with the average usability score.

### Logistical considerations

Logistical considerations largely centred around the chairs that would be used by the patients during the VR exercise experience (Fig. 3). When considering the infusion chairs they had experienced in the past, participants reported aspects such as back support, footrest position and correct height adjustment needed to be considered. When an alternative chair option was discussed, comments are centred around the need for arm rests, both to rest an arm for cannula safety and to provide stability and safety to the person while immersed.

Other logistical considerations centred around the need for flexibility in the delivery of the intervention to accommodate the fluctuating physical and emotional states of people receiving chemotherapy. For example, one participant reported “It would be good to have it as an option, like, “this is a good week, I can manage this”” (62-year-old female breast cancer survivor). Participants also mentioned other logistical considerations, such as the need for medical/oncologist approval prior to commencement, the impact of certain medications and the need to monitor the prescribed activity intensity.

## Discussion

This is the first study to explore the use of immersive VR combined with exercise during chemotherapy infusion. Overall, the participants’ experiences were positive, and all reported high perceived utility of immersive VR exercise if implemented during chemotherapy infusion. Participants reported overall low intensity of VR symptoms, average system usability and moderate presence. Possible barriers to use and logistical considerations were identified. Overall, the findings suggest that immersive VR exercise during infusion may be acceptable and worth pursuing as a supportive care intervention, and there may be potential psychological benefits beyond the proposed benefit of increased participation in physical activity.

Given the lack of evidence on combined immersive VR and exercise interventions during chemotherapy infusion, recommendations should be based on existing research examining stand-alone exercise or stand-alone VR interventions during chemotherapy. Stand-alone VR studies, which typically have a focus on distraction, relaxation and symptom mitigation, report favourable ease of use, satisfaction and desire to continue the intervention and little-to-no cybersickness or adverse events [27–29]. The qualitative and quantitative data in this study supports this, with overall low-intensity symptoms experienced by the participants, and subjective reporting of enjoyment of the experience. Overall, these studies suggest a beneficial impact on physical (e.g. nausea and fatigue), psychological and emotional symptoms (e.g. reduced fear and anxiety, and increased sense of control) and an altered perception of time (time went quicker) [27, 29, 30]. Workflow issues and delays in patient care related to issues with VR headset and setup were reported in one study [27]. Feasibility studies on exercise during infusion report these interventions to be safe with no adverse events; however, one study reported low uptake [17, 31]. Regarding future uptake, it is promising that all participants in this study reported they would have trialled the VR exercise experience if offered to them during chemotherapy. Given the paucity of evidence, studies focussed on implementation, service and client outcomes of combined immersive VR exercise during chemotherapy infusion are required to allow appropriate interventions and implementation strategies to be developed. Additionally, a recent review of VR use in mobility rehabilitation identified that 37% of studies employed off-the-shelf programmes [32] highlighting that many VR studies focus on whether the technology is effective, rather than a well-designed, domain-specific VR application, and generally provide little information about VR intervention design [33]. High-quality VR research is becoming increasingly important to understand how the relationship between the VR technology and VR application makes the intervention effective [34].

The findings from this study provide some important implementation considerations that need further investigation with cancer survivors currently receiving chemotherapy and other stakeholders such as infusion suite nurses. During discussion and objectively, most of the participants reported some short-lived symptoms because of their VR exercise experience, but this did not appear to impact their positivity regarding the potential utility and benefit of the experience in infusion suites expressed during interviews and focus groups. Strategies to mitigate other safety considerations such as chair logistics, risk of cannula dislodgement and claustrophobia will need further exploration, but adjustable height exercise equipment, gaze-controlled software (reducing arm movement) and the use of pass-through (to reduce presence and possible emotional responses) are some options [35].

There appear to be multiple possible benefits of engagement in immersive VR exercise during infusion. In the current study, the hypothesised benefit is the promotion of physical activity from an enjoyable exercise experience. The participants also identified potential benefits such as the sense of distraction, achievement, positivity and improved mental wellbeing, highlighting the potential for psychological benefits. Recent studies among university students suggest that combined VR exercise can promote positive mood states when compared to non-VR exercise [36] and potentially reduce anxiety and depression [37]. These findings should be interpreted cautiously due to low confidence in the evidence at this stage. Even though not yet reported during chemotherapy infusion, non-immersive VR exercise during in-centre haemodialysis with people diagnosed with kidney disease has been trialled [38]. The context, goals and volume of exercise delivered (e.g. per week) will differ considerably with chemotherapy-based programmes that are typically delivered fortnightly, compared to in-centre haemodialysis that is typically delivered three times a week. However, a reduction in depression and anxiety symptoms after 3 months of light VR exercise was reported [38]. The studies exploring stand-alone VR during infusion also support positive psychological outcomes, suggesting distraction leads to a reduction in anxiety and distress levels [29]. Given that these potential psychological benefits were similarly identified by cancer survivors, inclusion of psychological outcomes among the patient-centred outcomes evaluated in future studies is recommended.

## Limitations

The results were obtained from a small number of participants who, although they had not used VR before, reported high confidence and comfort with digital technology use. Thus, the findings are unlikely to be generalisable to all cancer survivors who have experienced chemotherapy and may not be representative of the full range of confidence and comfort with digital technology use expected to encounter in this population. Yet, there are data to suggest digital technology use is common among Australians aged 55 to 74 years, and this age range is common for people undergoing active cancer treatment and is reflective of the age of the sample [39].

The participants had experienced chemotherapy on average, 3.4 years earlier, suggesting their recall of the chemotherapy setting and experience could be limited and possibly distorted by time. Further, some logistical considerations and context-specific barriers may no longer be relevant, given the potential for advances/changes in that timeframe or may only be applicable to certain infusion suite sites. Inconsistencies across participants in the types of VR scenes experienced (i.e. animated vs real-life) and virtual travel issues may have influenced the subjective and objective reporting of the experience. Despite the software issues and resultant animated VR scenes, the two individuals reported average presence scores (4.5 and 4.33) and moderate to high usability scores (82.5 and 72.5) and identified they would have liked to use the experience during chemotherapy, whereas the one individual who experienced virtual travel issues reported low presence and usability (1.67 and 45), but still would have liked to try VR exercise during chemotherapy. The study also only involved potential end-users, while the perception of staff, such as infusion suite nurses who will likely deliver VR exercise interventions during infusion administration, did not participate. Staff perceptions across the entire service delivery chain need to be explored and considered to determine feasibility.

## Conclusion

This study is the first to explore the perceptions and utility of immersive VR exercise during chemotherapy infusion, and an initial step toward developing an immersive VR exercise experience for use during infusion. The findings suggest that VR exercise during infusion may be acceptable and worth pursuing as a possible supportive care intervention. Additionally, the selection of an appropriate VR application needs to be considered in conjunction with the VR implementation. Future studies should engage cancer survivors who are currently undergoing chemotherapy to contribute to co-design to enhance the patient experience and potentially improve health outcomes. In addition, stakeholders such as infusion suite management, oncologists and nursing staff should be involved to identify the multi-level factors that might affect implementation.

## Supplementary Information

Below is the link to the electronic supplementary material.

ESM 1 (PDF 70.7 KB)

## Data Availability

The data that support the findings of this study are not openly available due to reasons of sensitivity and are available from the corresponding author upon reasonable request.
