# Supplementary material for: Adult cancer survivors’ perceptions of immersive virtual reality exercise and its utility during chemotherapy infusion: a concurrent mixed method exploratory study
Source: Support Care Cancer. 2026 Jul 4;34(7):734. doi: 10.1007/s00520-026-10920-7 (PMC13332874; doi:10.1007/s00520-026-10920-7)
Supplement: Supplementary file 1 — (PDF 70.7 KB) [file 520_2026_10920_MOESM1_ESM.pdf]

Adult cancer survivors' perceptions of immersive virtual reality exercise and its utility during chemotherapy infusion

Journal of Supportive Care in Cancer

Katia Ferrar, Belinda Lange, Thomas Beltrame, Liz Buckley, Keng Hao Chew, Jonathan Foo, Max Hollis, Emma Kemp, Bogda Koczwara, Peggy Lim, David Mizrahi, Nicole May, Jessica Thomson, Joan Schumacher and David Hobbs.

Dr Katia Ferrar – Corresponding author

Flinders University, College of Medicine and Public Health, Flinders Health and Medical Research Institute, Bedford Park, South Australia, Australia

Email: katia.ferrar@flinders.edu.au

Supplementary Table 1: Codes, sub-categories and categories reflecting the discussions around the participants' VR exercise experience. Frequency of each code reported in brackets.

Codes (frequency reported)	Sub-categories	Category
Fun experience (6)<br>Pleasant (1)<br>Enjoyable (1)	Enjoyable	Positive VR exercise experiences
Didn't think about self (4)<br>VR curiosity (4)<br>Doing something positive (2)<br>Achievable (2)<br>Familiar experience (2)<br>No nausea (2)<br>Relaxing (1)<br>Motivating (1)<br>VR safety (1)<br>Sense of travel (1)		
Easy to use (1)<br>Good travel speed (1)<br>Pedals easy (1)<br>Good video quality (1)	Equipment-related	
Disorientation (1)<br>Increased heart rate (1)<br>Motion sick (1)<br>Nausea (1)	Short lived symptoms	Negative VR exercise experience
Unreal video (2)<br>Not like real bike (1)<br>Reduced reality (1)	Unrealistic	
Pedal resistance tricky (2)<br>Clunky pedal (4)<br>Pedal set up odd (1)	Pedal issues	
Poor quality video (3)<br>Software glitch (2)	Video quality issues	
Video pedal mismatch (4)<br>Bike safety anxiety (1)<br>Prefer outside exercise (1)<br>Moving too fast (1)		

Supplementary Table 2: Codes, sub-categories and categories reflecting the discussions around the utility of VR exercise during infusion. Frequency of each code reported in brackets.

Codes	Sub-categories	Category
Distraction (5)<br>Distraction from drugs (1)<br>Distraction from place (1)<br>Distraction from process (1)<br>Take out of environment (1)	Distraction from chemotherapy	Positive VR exercise outcomes
Exercise benefits chemo (3)<br>Exercise good during chemo (1)	Exercise good during chemo	
Feel on your own (4)<br>Sense of achievement (2)<br>Doing something positive (2)<br>Mental wellbeing (2)<br>Fitness (1)<br>Make time go faster (2)<br>Keep occupied (1)<br>Provide variety (1)<br>Stay awake (1)<br>All can use (1)<br>Feeling of control (1)		
Cold cap issues (3)<br>Unaware of surroundings (2)<br>Possible nausea/motion sick (2)<br>Cannular issues (1)<br>Physical restrictions (1)<br>Claustrophobia (1)<br>Miss the food trolley (1)<br>Headset issues (1)<br>Poisonous sweat (1)<br>Tech knowledge (1)		Possible barriers
Chair set up (2)<br>Appropriate chair (1)<br>Armrests required (3)<br>Adjustable chair (1)<br>Back support (1)<br>Foot rest (1)<br>Need safe chair (1)<br>Reach pedals (2)<br>Recumbent position (1)	Chair considerations	Logistical considerations
Ability will vary (3)<br>Flexible delivery required (2)	Flexible delivery	
Space (1)<br>Brain fog (1)<br>Headset ease of use (1)<br>Ability to move (2)<br>Need to monitor intensity (2)<br>Get accustomed (2)<br>Medical clearance (2)<br>Medications (2)<br>Negative associations (1)<br>No issues (1)<br>Good if on own (1)		
